# High Cytoplasmic FOXO1 and pFOXO1 Expression in Astrocytomas Are Associated with Worse Surgical Outcome

**DOI:** 10.1371/journal.pone.0069260

**Published:** 2013-07-09

**Authors:** Chao Chen, Tao Xu, Jinxu Zhou, Yong Yan, Weiqing Li, Hongyu Yu, Guohan Hu, Xuehua Ding, Juxiang Chen, Yicheng Lu

**Affiliations:** 1 Department of Neurosurgery, Shanghai Institute of Neurosurgery, Changzheng Hospital, Second Military Medical University, Shanghai, China; 2 Department of Pathology, Changzheng Hospital, Second Military Medical University, Shanghai, China; University of Portsmouth, School of Pharmacy & Biomedical Sciences, United Kingdom

## Abstract

FOXO1 is at a convergence point of receptor tyrosine kinase (RTK) signaling, which is one of the three core pathways implicated in glioblastoma. It was recently shown that FOXO1 can effectively induce glioma cell death and inhibit tumor growth through cell cycle arrest and apoptosis. We therefore evaluated FOXO1 and pFOXO1 protein expression in 181 primary astrocytoma samples and 16 normal brain samples. Astrocytoma samples expressed higher cytoplasmic FOXO1 and pFOXO1 than normal brain samples. Nuclear pFOXO1 level was significantly higher than nuclear FOXO1 in astrocytomas. High cytoplasmic FOXO1 expression was associated with older onset age (P = 0.001) and higher WHO grade (P = 0.001). The trend was also observed between cytoplasmic pFOXO1 expression and WHO grade although not significant. Univariate survival analysis showed that both high cytoplasmic FOXO1 and pFOXO1 expression indicated a significantly shorter median overall survival and progression-free survival. Multivariate survival analysis revealed cytoplasmic FOXO1 expression, cytoplasmic pFOXO1 expression, WHO grade, gender, extent of resection and radiotherapy to be independent prognostic factors for overall survival and progression-free survival. Thus, our data suggested that cytoplasmic FOXO1 and pFOXO1 expression may serve as valuable prognostic variables in astrocytomas and may have significant implications for the development and application of targeted therapy.

## Introduction

Glioma is the most common primary type of brain tumor, with an incidence rate of about six per 100,000 per year worldwide [1]. About 70% of newly diagnosed gliomas are malignant. Despite multimodality therapy including maximal resection and adjuvant chemotherapy and radiotherapy, the overall outcome of patients with malignant glioma remains dismal. The median survival is about 12–15 months for patients with glioblastoma multiforme and 5-year survival rate is less than 10% [2], [3].

To understand the underlying molecular pathogenesis of glioblastoma, The Cancer Genome Atlas (TCGA) studied 206 glioblastoma samples using microarray technology and the analyses identified three core signal pathways implicated in glioblastoma, including receptor tyrosine kinase (RTK) signaling, and the P53 and RB tumor suppressor pathways [4].

FOXO (Forkhead box, class O), which is a subfamily of forkhead transcription factor, is at the convergence point of RTK signaling. The FOXO family consists of FOXO1 (also known as FKHR), FOXO3a, FOXO4 and FOXO6. FOXO1 is considered as a tumor repressor as it promotes cell-cycle arrest and apoptosis by regulating specific gene-expression programs. Activation of FOXO1 results in upregulation of the cyclin-dependent kinase inhibitor p27 and downregulation of D-type cyclins, thereby arresting the cell cycle at the G1 phase[5]–[7]. Activation of FOXO1 also increases the transcription and half-life of cyclin-dependent kinase inhibitor p27^KIP1^. FOXO1 triggers apoptosis through regulation a number of proapoptotic proteins, including Bim and TRAIL [8], [9]. In both p53-deficient and p53-proficient cells, silencing of FOXO1 dimishes DNA damage-induced cell death [10]. Besides working as a transcription factor, cytoplasmic FOXO1 binds and activates the autophagy-regulating protein, Atg7 and is involved in stress-induced autophagy in cancer cells, which results in anti-neoplastic effect. This function is fully independent of its transcriptional role [11], [12]. In glioma, constitutive nuclear FOXO1 expression can induce cell death *in vitro* and prolong survival *in vivo* in xenograft models [13].

Phosphorylation plays a central role for regulation of FOXO1 function [14]. In the presence of growth factor signaling, FOXO1 is phosphorylated by Akt in two or three conserved residues (T24, S256, and S319) [15], that is followed by their interaction with 14-3-3 proteins and nuclear exclusion [16]. Cytoplasmic FOXO1 is inactive in transcriptional function, which results in abrogation of proapoptotic function and cell cycle regulation [17].

Clinically, FOXO1 phosphorylation has been associated with disease progression in several cancers, including leukemia [18], alveolar rhabdomyosarcoma [19], prostate cancer [20], gastric cancer [21] and soft tissue sarcoma [22], but its clinical and pathologic significance in glioma has not been investigated yet. In this study, we examined expression of FOXO1 and pFOXO1 protein in a large cohort of astrocytomas using tissue microarray (TMA) technology and analyzed for their correlations with clinical characteristics as well as disease progression.

## Materials and Methods

### Patients and Samples

This study evaluated histologic sections from 190 patients with different grades of astrocytoma undergoing surgical resections in the department of Neurosurgery, Changzheng Hospital, Shanghai, China between 1999 and 2008. Both the patients and next of kin were asked for permission with written informed consent of operation. The selection criteria of this study were as follows: (i) the subject had a primary diagnosis of astrocytoma and no history of other tumors; (ii) the subject had complete clinical data, including age, gender, clinical manifestations, mean tumor diameter (MTD, defined as the geometric mean of the 3 diameters on MRI scan), extent of resection, histological type, pathological grade and adjuvant therapy; (iii) the subject underwent evaluation by enhanced head MRI scans for tumor relapse or progression after surgery at least once every six months. Overall survival (OS) was defined as the time between diagnosis and death and progression-free survival (PFS) was defined as the time between diagnosis and the date of documented tumor recurrence or further growth of residual tumor detected by enhanced MRI scan. Sixteen normal brain tissues were obtained from surgical resections of trauma patients, for whom a partial resection of normal brain tissue was required as decompression treatment for their severe head injuries to reduce increased intracranial pressure. As these patients were unconscious, next of kin were asked for permission with written informed consent of operation. The tissue microarray (TMA) was constructed based on these samples. The study protocol was approved by Tissue Committee and Research Ethics Board of Second Military Medical University.

### TMA Construction and Immunohistochemical Analysis

Tissue microarray was constructed in Shanghai Biochip Co Ltd, according to the protocol [23]. After verification with hematoxylin and eosin (H&E) staining, areas showing the histopathologic features of tumor or normal cortex tissue were selected on slides. And then representative areas were marked on the corresponding paraffin block for TMA construction. One core punch sample was taken from each specimen, measuring 1.5 mm in the greatest dimension from the center for tumor loci. Anti-FOXO1 polyclonal antibody (1∶100, ab39670; Abcam, UK) and anti-pFOXO1 (pFOXO1; Ser256) polyclonal antibody (1∶200, ab38501; Abcam, UK) were used as the primary antibody. Immunohistochemical staining was performed using a streptavidin peroxidase procedure (avidin-biotin complex method) after antigen retrieval using an autoclave. The sections not incubated with the primary antibody were used as negative controls and sections from a patient of non-small cell lung cancer were used as positive controls.

The evaluation of immunohistochemical results of TMA was performed by two independent pathologists with no knowledge of the clinical data. Five characteristic microscopic fields (×400) were randomly selected for each patient. The density of positive staining was scored using the scale from 0 to 3 (0 for no immunostaining, 1 for light-brown color, 2 for medium-brown color, and 3 for dark-brown color), while the percentage of positive staining area was scored from 0 (complete absence) to 100 (all cells labeling). The labeling intensity and labeling percentage generated a histology score (H-score) ranging from 0 to 300, with H-score = intensity of immunolabel (range, 0–3)×the percentage of cancer cells that were reactive [24]. All discrepancies in scoring were reviewed, and a consensus was reached. The median H-score of all samples was used as cut-off for dividing the overexpression and low-expression groups. In the case of nuclear and cytoplasmic expression, both nuclear and cytoplasmic staining signals were interpreted and recorded separately.

### Statistical Analysis

For comparison of the expression level of FOXO1 and pFOXO1 protein between different groups (i.e. astrocytoma versus normal brain tissues), Mann-Whitney U test was used. The correlations of FOXO1 and pFOXO1 expression were analyzed with the Spearman correlation test. For the association between FOXO1 and pFOXO1 expression and clinicopathological factors, either the χ2 test or the Fisher’s exact test (two-sided) was performed. Cumulative survival time was calculated by the Kaplan-Meier method and analyzed by the log-rank test. Multivariate analyses were performed by Cox regression model. All statistical analyses were performed with SPSS version 18.0 software (SPSS Inc, Chicago, USA) and P<0.05 was considered as significant difference in statistical between groups.

## Results

### Basic Characteristics of Tissue Microarray

The original database contained 190 astrocytoma samples and 16 normal brain tissue samples. Seven samples were disqualified because of tissue damage or loss. Another 2 samples were disqualified for no adequate tumor tissues. 181 astrocytoma samples (61 grade II diffuse astrocytomas, 27 grade III anaplastic astrocytomas and 93 grade IV glioblastomas) and 16 normal brain samples were qualified for evaluation. Mean age of trauma patients whose normal brain tissue samples were obtained was 41.7 years (range, 22 to 68 years). The basic clinical pathologic characteristics of astrocytoma patients list in Table 1.

**Table 1:** Clinio-pathological characteristics of 181 patients of astrocytomas.

Variable	Histological Classification (WHO)		
	Grade II	Grade III	Grade IV
Number of patients	61	27	93
Age (Years)			
≤50	54	16	32
>50	7	11	61
Gender			
Male	46	16	62
Female	15	11	31
Seisure			
No	40	25	84
Yes	21	2	9
IICP			
No	46	16	50
Yes	15	11	43
MTD			
<5 cm	32	10	38
≥5 cm	29	17	55
Extent of surgery			
Total	49	22	69
Sub-total	9	1	21
Partial	3	1	3
Biopsy	0	3	0
Radiotherapy			
Yes	33	18	67
No	28	9	26
Chemothearpy			
Yes	33	17	70
No	28	10	23
Follow-up (months)			
Range	0–114 (n = 56)*	3–80 (n = 25)*	0–102 (n = 89)*
Median	73	28	12
Recurrence	44.6%	84.0%	94.4%
Death	39.3%	76.0%	91.0%

Abbreviations: IICP, increased intracranial pressure; MTD, mean tumor diameter.

* The survival data of 11 patients, including 5 diffuse astrocytomas (Grade II), 2 anaplastic astrocytomas (Grade III) and 4 glioblastomas (Grade IV) was not available.

### Expression of FOXO1 Protein in Astrocytomas and Normal Brain Tissues

**Figure 1:**
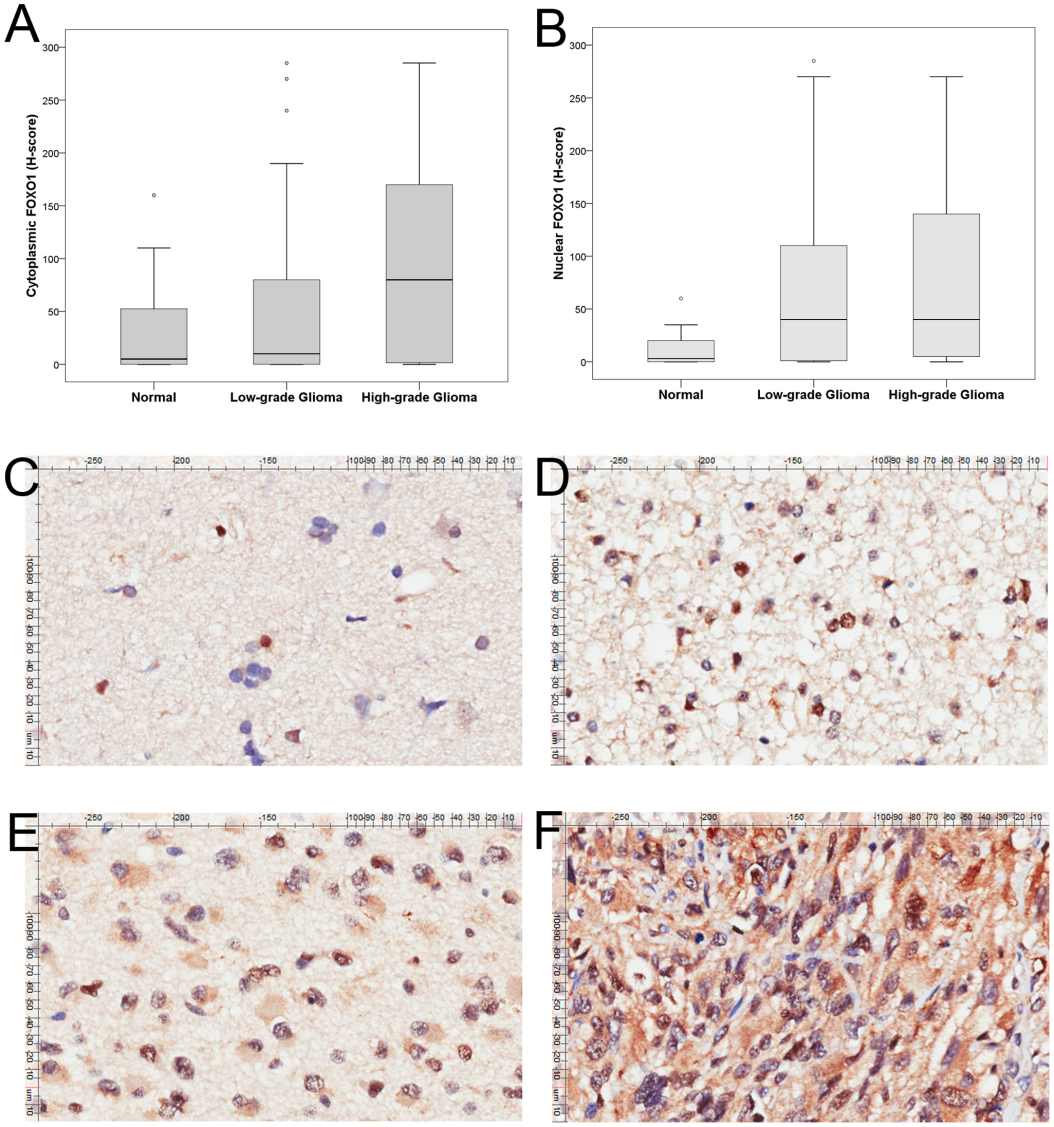
Tissue microarray analysis of FOXO1 expression. (A, B) Cytoplasmic and nuclear FOXO1 expressions were higher in astrocytomas than in normal brain tissues. High-grade gliomas express more cytoplasmic FOXO1 than low-grade gliomas while nuclear FOXO1 expression was comparable in both. The horizontal line inside the box represents the median. The outliers are cases with the values between 1.5 and 3 box-lengths from the 75th percentile or 25th percentile. Representative images of FOXO1 expression in normal brain tissue (C, ×200); grade II diffuse astrocytoma (D, ×200); grade III anaplastic astrocytoma (E, ×200) and grade IV glioblastoma (F, ×200).

FOXO1 protein expression was located in either cytoplasm or nucleus or both (Fig. 1C–F). The median H-score for cytoplasmic FOXO1 was 55 (range, 0–285) in astrocytoma samples, which was significantly higher than that of normal brain tissue samples (median H-score 5, range 0–160) (P = 0.036). Nuclear FOXO1 expression in astrocytoma cells (median H-score 40, 0–285) was also significantly higher than normal brain tissue (median H-score 3, 0–60) (P = 0.001). Cytoplasmic FOXO1 expression in high-grade glioma was significantly higher than that of low-grade glioma (median H-score: 80 versus 10, P = 0.005) while nuclear FOXO1 expression was comparable in both (median H-score: 40 versus 40, P = 0.471) (Fig. 1A, B).

### Expression of pFOXO1 Protein in Astrocytomas and Normal Brain Tissues

**Figure 2:**
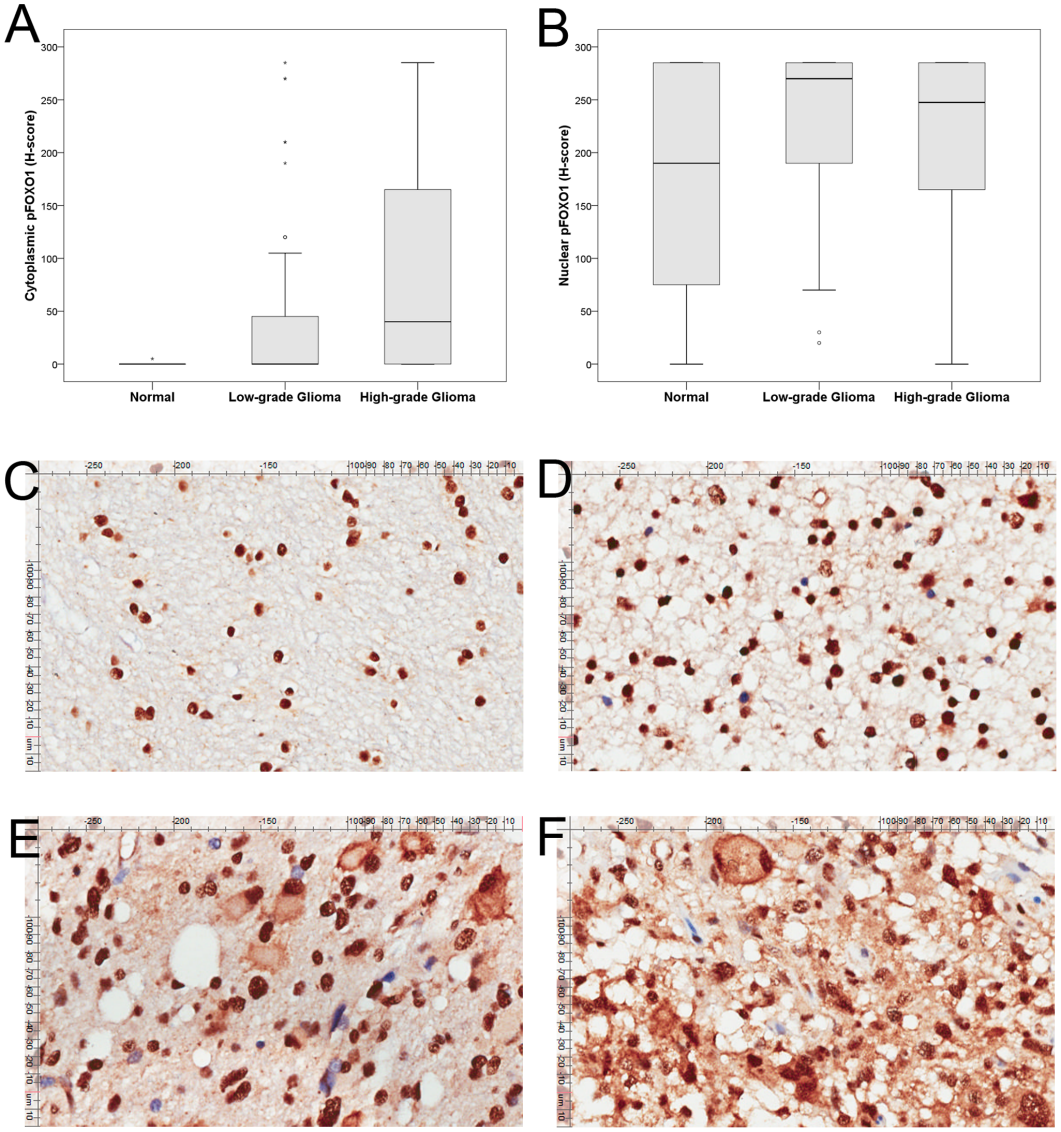
Tissue microarray analysis of pFOXO1 expression. (A, B) Cytoplasmic pFOXO1 expressions was higher in high-grade astrocytomas than low-grade and normal brain tissues. Nuclear pFOXO1 expression was comparable in three group. The horizontal line inside the box represents the median. The outliers are cases with the values between 1.5 and 3 box-lengths from the 75th percentile or 25th percentile. Representative images of pFOXO1 expression in normal brain tissue (C, ×200); grade II diffuse astrocytoma (D, ×200); grade III anaplastic astrocytoma (E, ×200) and grade IV glioblastoma (F, ×200).

pFOXO1 protein expression was strongly present in nucleus in normal brain tissues and mostly negative in cytoplasm (Fig. 2C). For astrocytomas, pFOXO1 expression was located in either cytoplasm or nucleus or both, but strongly present in nucleus (Fig. 2D–F). The median H-score for cytoplasmic pFOXO1 was 40 (range, 0–285) in high-grade astrocytoma samples, which was significantly higher than that of low-grade astrocytomas (median H-score 0, 0–285) (P = 0.001, Fig. 2A). Nuclear pFOXO1 expression was comparable in normal brain tissue (median H-score 190, 0–285), low-grade astrocytoma (median H-score 270, 20–285) and high-grade astrocytoma (median H-score 247.5, 0–285) (P = 0.108, Fig. 2B).

There was a significant correlation between FOXO1 and pFOXO1 expression (cytoplasmic FOXO1 versus pFOXO1, *ρ* = 0.837, P<0.001; nuclear FOXO1 versus pFOXO1, *ρ* = 0.151, P = 0.034). Nuclear pFOXO1 level was significantly higher than that of FOXO1, which indicated the phosphorylation of FOXO1 is probably critical in the genesis of astrocytomas.

### Correlation of FOXO1 and pFOXO1 Protein Expression with Clinical Characteristics in Astrocytomas

The relationship between FOXO1 and pFOXO1 protein expression in tumor tissues and other clinico-pathological characteristics is shown in Table 2 and Table S1. High cytoplasmic FOXO1 expression (H-score ≥60) was significantly associated with older onset age (P = 0.001) and higher WHO grade (P = 0.001). A trend was observed between high cytoplasmic FOXO1 expression and increased intracranial pressure (IICP) although not significant (P = 0.082). High cytoplasmic pFOXO1 expression was also related with higher WHO grade although not significant (P = 0.102) (Table 2). Thus, both high cytoplasmic expression of FOXO1 and pFOXO1 were associated with malignant characteristics of astrocytomas. Nuclear pFOXO1 expression was significantly associated with larger mean tumor diameter (MTD) (P = 0.015) and nuclear FOXO1 expression was not associated with these clinico-pathological characteristics (Table S1).

**Table 2:** Association between cytoplasmic FOXO1 and pFOXO1 expression and clinico-pathological parameters.

Variables	N	High Cystoplasmic FOXO1 expression		P-value	High Cytoplasmic pFOXO1 expression		P-value
		Number (%)	Odds ratio (95%CI)		Number (%)	Odds ratio (95%CI)	
Age (Years)							
≤50	102	40 (39.2%)	1	**0.001**	39 (38.2%)	1	0.066
>50	79	50 (63.3%)	2.67 (1.46–4.90)		41 (51.9%)	1.74 (0.96–3.16)	
Gender							
Male	124	57 (46.0%)	1	0.136	55 (44.4%)	1	0.980
Female	57	33 (57.9%)	1.62 (0.86–3.05)		25 (43.9%)	0.98 (0.52–1.84)	
Seisure							
No	149	77 (51.7%)	1	0.257	67 (45.0%)	1	0.654
Yes	32	13 (40.6%)	0.64 (0.30–1.39)		13 (40.6%)	0.84 (0.39–1.82)	
IICP							
No	112	50 (44.6%)	1	0.082	52 (46.4%)	1	0.442
Yes	69	40 (58.0%)	1.71 (0.93–3.14)		28 40.6%)	0.79 (0.43–1.45)	
MTD							
<5 cm	80	42 (52.5%)	1	0.506	34 (42.5%)	1	0.682
≥5 cm	101	48 (47.5%)	0.82 (0.46–1.48)		46 (45.5%)	1.13 (0.63–2.04)	
WHO grade							
II	61	21 (34.4%)	1	**0.001**	21 (34.4%)	1	0.102
III	27	10 (37.0%)	1.12 (0.44–2.88)		11 (40.7%)	1.31 (0.52–3.33)	
IV	93	59 (63.4%)	3.30 (1.68–6.50)		48 (44.2%)	2.03 (1.04–3.96)	

Abbreviations: IICP, increased intracranial pressure; MTD, mean tumor diameter.

### FOXO1 and pFOXO1 Protein Expression as Prognostic Factors in Astrocytomas

170 astrocytoma patients were available for survival analysis (the survival data of 11 patients, including 5 diffuse astrocytomas, 2 anaplastic astrocytomas and 4 glioblastomas was not available).

**Figure 3:**
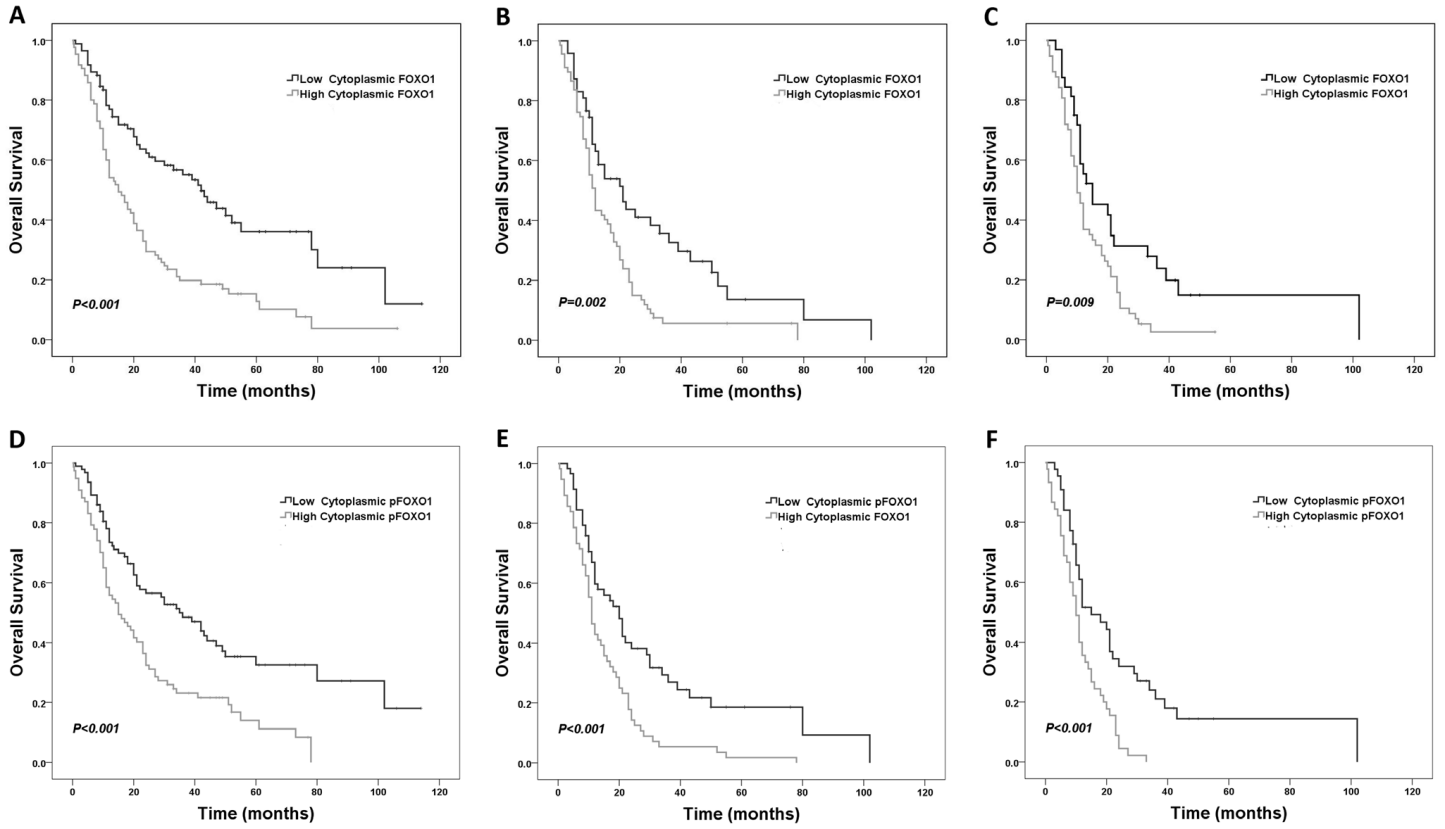
Association of cytoplasmic FOXO1 and pFOXO1 expression with overall survival in astrocytoma patients. Kaplan-Meier survival curves comparing high and low cytoplasmic FOXO1/pFOXO1 expression are shown. (A, D) all astrocytoma patients; (B, E) high-grade glioma (anaplastic astrocytoma and glioblastoma multiforme) patients; (C, F) glioblastoma multiforme patients. FOXO1: A–C; pFOXO1: D–F.

Patients with high cytoplasmic FOXO1 expression had a significantly shorter median OS and PFS than those with low cytoplasmic FOXO1 expression (OS: 15 months versus 42 months, PFS: 12 months versus 34 months) (Fig. 3A, S1A). High cytoplasmic pFOXO1 expression was also significantly associated with shorter median OS and PFS (OS: 15 months versus 35 months, PFS: 12 months versus 29 months) (Fig. 3D, S1D). Univariate analysis of other clinico-pathological variables revealed that older onset age (P<0.001), presence of seizure (P = 0.011), small extent of resection (P = 0.001) and higher WHO grade (P<0.001) were statistically correlated with shorter OS.

**Figure 4:**
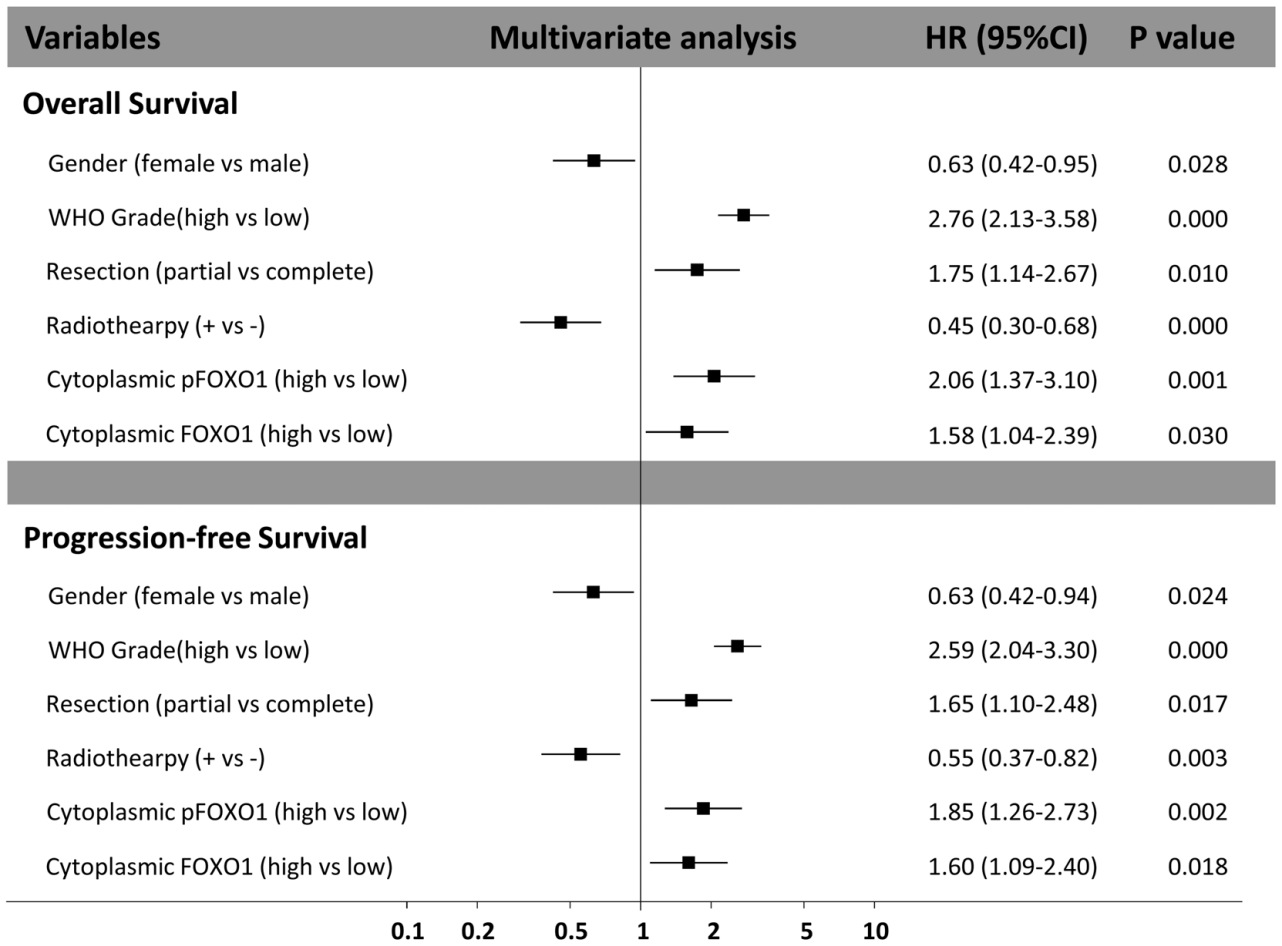
Multivariate analysis of factors associated with survival and progression in all astrocytoma patients.

The multivariate Cox regression analysis indicated that male gender, higher tumor grade, partial resection, no adjuvant radiotherapy, high cytoplasmic FOXO1 expression, and high cytoplasmic pFOXO1 expression were independent and significant risk factors of poor prognosis in astrocytoma patients for both OS and PFS. Besides tumor grade, cytoplasmic pFOXO1 expression level had the greatest hazard ratio (HR) value for survival (HR 2.06, 95% CI 1.37–3.10; P = 0.001) and recurrence (HR 1.85, 95% CI 1.26–2.73; P = 0.002) (Fig. 4). The hazard ratio (HR) value of cytoplasmic FOXO1 is 1.58 (95% CI 1.04–2.39; P = 0.030) for survival and 1.60 (95% CI 1.09–2.40; P = 0.018) for recurrence (Fig. 4).

Nuclear FOXO1 and pFOXO1 expression were not associated with prognosis of astrocytoma patients in this group.

### Impact of FOXO1 and pFOXO1 Expression on Prognosis of High-grade Glioma (HGG)

Since high-grade glioma (HGG) patients had much lower survival expectancy after surgery than low-grade glioma (LGG), we performed sub-group analysis to investigate FOXO1 and pFOXO1 expression on prognosis of the group of patients with dismal prognosis.

As shown in Fig. 3B and S1B, high cytoplasmic FOXO1 expression was correlated with shorter median OS and PFS compared with low expression in HGG (OS: 12 months versus 21 months, PFS: 9 months versus 20 months). And the association was also applied for high cytoplasmic pFOXO1 expression (OS: 11 months versus 20 months, PFS: 8 months versus 17 months) (Fig. 3E, S1E).

When specific to glioblastoma, both high cytoplasmic FOXO1 and pFOXO1 expression were associated with shorter OS and PFS (OS: 10 months versus 15 months for both, PFS: 8 months versus 15 months for FOXO1 and 7 months versus 12 months for pFOXO1 respectively) (Fig.3C, 3F, S1C, S1F).

The multivariate Cox regression analysis revealed that high cytoplasmic pFOXO1 expression was an independent prognosis factor for survival in both HGG and GBM (HGG: HR 2.35, 95%CI 1.54–3.59; P<0.001, GBM: HR 2.32, 95% CI 1.44–3.73; P<0.001) while cytoplasmic FOXO1 expression was not.

## Discussion

Despite recent advances in surgery, radiotherapy and chemotherapy, the prognosis of malignant gliomas has changed very little over the past two decades [2], [3], [25]. The comprehensive research by TCGA found three core pathways in glioblastoma and provided more therapeutic targets to overcome the plateau in treatment [4]. FOXOs are at the convergence point of RTK signaling which is a core pathway in glioblastoma, so it is interesting and meaningful to investigate their expression and impacts on prognosis. Our study analyzed the expression of FOXO1 and pFOXO1 protein and their biological significance in human astrocytoma for the first time, which demonstrated that high cytoplasmic FOXO1 and pFOXO1 expression were associated with higher WHO grade and a worse prognosis. In multivariate analysis, high cytoplasmic FOXO1 and pFOXO1 expression were independent prognostic factors for astrocytomas.

Due to the following aspects, FOXO1 and pFOXO1 could be good prognosis factors in astrocytomas. (1) FOXO1 regulates a number of cellular processes such as apoptosis, cell cycle arrest and autophagy that are highly relevant to cancer. It has been demonstrated that targeting FOXO1 can effectively induce glioma cell death *in vitro* and inhibit tumor growth *in vivo* [13]. Phosphorylation of FOXO1 and cytoplasmic sequestration would disrupt its proapoptotic function and regulation of cell cycle. (2) PI3K-Akt in RTK signaling is the main upstream mediator of phosphorylation for FOXO1, which indicated that subcellular distribution and phosphorylation level of FOXO1 can reflect the activity of RTK signaling. The P53 tumor suppressor pathway, another critical pathway involved in glioblastoma, is also involved in FOXO1 regulation. Once in the cytoplasm, FOXO1 is ubiquitinated and subjected to degradation by the proteasome. MDM2, downstream of P53, acts as an ubiquitin E3 ligase to regulate the degradation of FOXO1 [26]. High cytoplasmic FOXO1 and pFOXO1 expression may be partially contributed by low activity of the P53 pathway. (3) FOXO1 is involved in chemoresistance through enhancing the expression of several key enzymes in the antioxidant defense system, including DNA repair enzyme GADD45α, mitochondrial MnSOD and catalase [27]–[29]. Cytoplasmic FOXO1 overexpression was frequently observed in ovarian cancer tissue samples from chemoresistant patients compared to chemosensitive patients [28].

Our study showed that higher cytoplasmic FOXO1 and pFOXO1 protein expression correlate with poor survival of astrocytoma patients, which is consistent with its inactivation by phosphorylation and cytoplasm translocation. Whether the association is partially contributed by its involvement in chemoresistance as ovarian cancer seems to be interesting to investigate. A similar result has been reported in breast cancer that cytoplasmic expression of FOXO3 was related to shorter overall survival in patients [30].

RTK signaling was shown to be altered in 88% of glioblastoma, underscoring its importance in gliomagenesis [4]. But targeted therapies that inhibit single RTKs, such as EGFR tyrosine kinase inhibitor gefitinib, did not show significant improvement in OS or PFS [31]. The poor efficacy of single treatments may be explained by concurrent activation of multiple RTKs in glioblastoma, including EGFR, ERBB2, PDGFRA and MET [32]. Considering FOXO1’s antiproliferative and proapoptotic effects and its convergence point in RTK signaling, targeting FOXO1 may be a solution of the dilemma. In vivo experiments have shown intratumoral injection with AdFOXO1; AAA (a mutant FOXO1 which cannot be negatively regulated by PI3K-Akt phosphorylation) can improve survival of mice implanted with glioma cell in brain [13]. Our study highlighted nuclear exclusion of FOXO1 in glioma progression. Combination therapy in which both FOXO1 phosphorylation and nuclear exclusion are inhibited may achieved better results. Semisynthetic leptomycin B (LMB), which could inhibit nuclear exclusion of FOXO1, showed significant antitumor effect in multiple mouse xenograft models [33]. Several other compounds targeting nuclear exclusion also have been found through high-throughput screens and showed antitumor efficacy [34]–[36].

In conclusion, our study demonstrated that cytoplasmic expression of FOXO1 and pFOXO1 are elevated in astrocytomas and are increased with higher grade. Moreover, both high cytoplasmic FOXO1 and pFOXO1 are independent prognosis factors for astrocytoma patients. However, due to potential artifact in the immunohistochemical study, the results should be interpreted with caution and further molecular studies of FOXO1 in gliomagenesis and chemoresistance are needed.

## Supporting Information

Figure S1 **Association of cytoplasmic FOXO1 and pFOXO1 expression with progression-free survival in astrocytoma patients.** Kaplan-Meier survival curves comparing high and low cytoplasmic FOXO1/pFOXO1 expression are shown. (A, D) all astrocytoma patients; (B, E) high-grade glioma (anaplastic astrocytoma and glioblastoma multiforme) patients; (C, F) glioblastoma multiforme patients. FOXO1: A–C; pFOXO1: D–F.
(TIF)

Table S1 **Association between nuclear FOXO1 and pFOXO1 expression and clinic-pathological parameters.**
(DOCX)
