# Supplementary figures and images for: High Cytoplasmic FOXO1 and pFOXO1 Expression in Astrocytomas Are Associated with Worse Surgical Outcome
Source: PLoS One. 2013 Jul 9;8(7):e69260. doi: 10.1371/journal.pone.0069260 (PMC3706417; doi:10.1371/journal.pone.0069260)

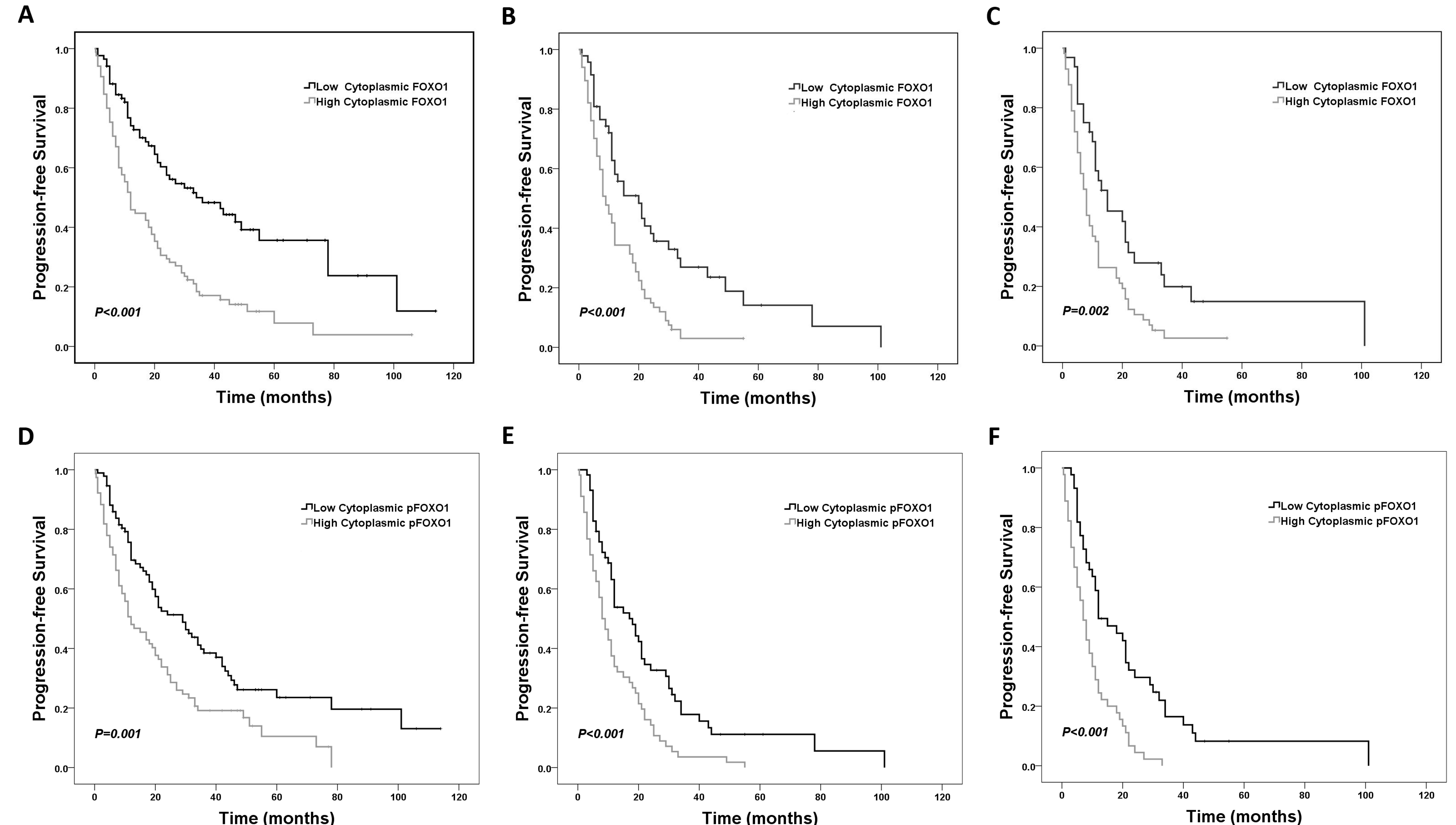

Supplement: Figure S1 — Association of cytoplasmic FOXO1 and pFOXO1 expression with progression-free survival in astrocytoma patients. Kaplan-Meier survival curves comparing high and low cytoplasmic FOXO1/pFOXO1 expression are shown. (A, D) all astrocytoma patients; (B, E) high-grade glioma (anaplastic astrocytoma and glioblastoma multiforme) patients; (C, F) glioblastoma multiforme patients. FOXO1: A–C; pFOXO1: D–F. (TIF) [file pone.0069260.s001.tif]
