# Supplementary material for: High Cytoplasmic FOXO1 and pFOXO1 Expression in Astrocytomas Are Associated with Worse Surgical Outcome
Source: PLoS One. 2013 Jul 9;8(7):e69260. doi: 10.1371/journal.pone.0069260 (PMC3706417; doi:10.1371/journal.pone.0069260)
Supplement: Table S1 — Association between nuclear FOXO1 and pFOXO1 expression and clinic-pathological parameters. (DOCX) [file pone.0069260.s002.docx]

**Table S1. Association between nuclear FOXO1 and pFOXO1 expression and clinic-pathological parameters.**

| **Variables** |  | **N** | **High Nuclear FOXO1 expression** | |  | **P-value** |  | **High Nuclear pFOXO1 expression** | |  | **P-value** |
| --- | --- | --- | --- | --- | --- | --- | --- | --- | --- | --- | --- |
|  |  |  | **Number (%)** | **Odds ratio (95%CI)** |  |  |  | **Number (%)** | **Odds ratio (95%CI)** |  |  |
| Age (Years) |  |  |  |  |  |  |  |  |  |  |  |
| ≤50 |  | 102 | 49 (48.0%) | 1 |  | 0.307 |  | 48 (47.1%) | 1 |  | 0.842 |
| >50 |  | 79 | 44 (55.7%) | 1.36 (0.75-2.45) |  |  |  | 36 (45.6%) | 0.94 (0.52-1.70) |  |  |
| Gender |  |  |  |  |  |  |  |  |  |  |  |
| Male |  | 124 | 62 (50.0%) | 1 |  | 0.583 |  | 54 (43.5%) | 1 |  | 0.255 |
| Female |  | 57 | 31 (54.4%) | 1.19 (0.64-2.24) |  |  |  | 30 (52.6%) | 1.44 (0.77-2.70) |  |  |
| Seisure |  |  |  |  |  |  |  |  |  |  |  |
| No |  | 149 | 77 (51.7%) | 1 |  | 0.863 |  | 67 (45.0%) | 1 |  | 0.401 |
| Yes |  | 32 | 16 (50.0%) | 0.94 (0.44-2.01) |  |  |  | 17 (53.1%) | 1.39 (0.65-2.98) |  |  |
| IICP |  |  |  |  |  |  |  |  |  |  |  |
| No |  | 112 | 58 (51.8%) | 1 |  | 0.890 |  | 49 (43.8%) | 1 |  | 0.361 |
| Yes |  | 69 | 35 (50.7%) | 0.96 (0.53-1.75) |  |  |  | 35 (50.7%) | 1.32 (0.73-2.42) |  |  |
| MTD |  |  |  |  |  |  |  |  |  |  |  |
| <5cm |  | 80 | 39(48.8%) | 1 |  | 0.528 |  | 29(36.3%) | 1 |  | **0.015** |
| ≥5cm |  | 101 | 54 (53.5%) | 1.21 (0.67-2.17) |  |  |  | 55 (54.5%) | 2.10 (1.15-3.84) |  |  |
| WHO grade |  |  |  |  |  |  |  |  |  |  |  |
| II |  | 61 | 31 (50.8%) | 1 |  | 0.691 |  | 33 (54.1%) | 1 |  | 0.272 |
| III |  | 27 | 12 (44.4%) | 0.77 (0.31-1.92) |  |  |  | 10 (37.0%) | 0.50 (0.20-1.26) |  |  |
| IV |  | 93 | 50 (53.8%) | 1.13 (0.59-2.15)) |  |  |  | 41 (44.1%) | 0.67 (0.35-1.28) |  |  |

Abbreviations: IICP, increased intracranial pressure; MTD, mean tumor diameter.
